# Bis[2-(1*H*-pyrazol-3-yl-κ*N* ^2^)pyridine-κ*N*]dithio­cyanato-κ*N*,κ*S*-cadmium(II)

**DOI:** 10.1107/S1600536810034604

**Published:** 2010-09-04

**Authors:** Hua Cai, Ying Guo, Jian-Gang Li

**Affiliations:** a College of Science, Civil Aviation University of China, Tianjin 300300, People’s Republic of China

## Abstract

The mol­ecular structure of the mononuclear complex, [Cd(SCN)_2_(C_8_H_7_N_3_)_2_], contains a Cd^II^ atom in a distorted octa­hedral coordination defined by five N atoms from two bidentate chelate 2-(1*H*-pyrazol-3-yl)pyridine ligands and by one SCN^−^ anion. The second SCN^−^ anion provides its S atom for completion of the coordination sphere. The complex is linked to four others by N—H⋯N and N—H⋯S hydrogen-bonding inter­actions between the pyrazol N—H group and the terminal S and N atoms of neighbouring SCN^−^ anions. This arrangement leads to the formation of sheets parallel to (100). Face-to-face π–π stacking inter­actions with shortest inter­planar distances of 3.805 (2) and 3.696 (2) Å help to consolidate the crystal packing.

## Related literature

For background to self assembly in supra­molecular chemistry, see: Beatty (2003 ▶); Braga *et al.* (2003 ▶); Chen & Liu (2002 ▶); Zhang *et al.* (2004 ▶). For related structures, see: Hu *et al.* (2008 ▶).
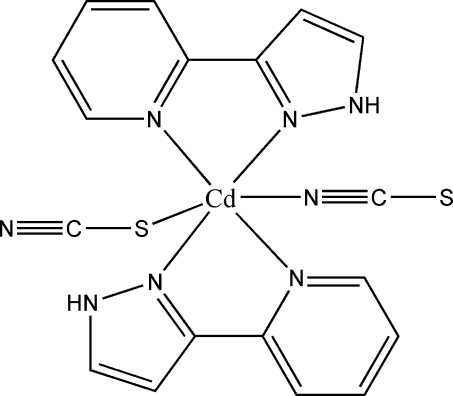


## Experimental

### 

#### Crystal data

[Cd(NCS)_2_(C_8_H_7_N_3_)_2_]
*M* *_r_* = 518.89
Monoclinic, 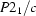

*a* = 14.4612 (19) Å
*b* = 9.6043 (12) Å
*c* = 14.9089 (19) Å
β = 99.290 (2)°
*V* = 2043.5 (5) Å^3^
*Z* = 4
Mo *K*α radiation
μ = 1.30 mm^−1^
*T* = 296 K
0.32 × 0.26 × 0.22 mm

#### Data collection

Bruker APEXII CCD area-detector diffractometer
Absorption correction: multi-scan (*SADABS*; Bruker, 2003 ▶) *T*_min_ = 0.682, *T*_max_ = 0.764
10166 measured reflections
3602 independent reflections
3119 reflections with *I* > 2σ(*I*)
*R*_int_ = 0.022

#### Refinement

*R*[*F* ^2^ > 2σ(*F* ^2^)] = 0.023
*wR*(*F* ^2^) = 0.053
*S* = 1.05
3602 reflections
262 parameters
H-atom parameters constrained
Δρ_max_ = 0.33 e Å^−3^
Δρ_min_ = −0.24 e Å^−3^

### 

Data collection: *APEX2* (Bruker, 2003 ▶); cell refinement: *SAINT* (Bruker, 2003 ▶); data reduction: *SAINT*; program(s) used to solve structure: *SHELXS97* (Sheldrick, 2008 ▶); program(s) used to refine structure: *SHELXL97* (Sheldrick, 2008 ▶); molecular graphics: *SHELXTL* (Sheldrick, 2008 ▶) and *DIAMOND* (Brandenburg, 2006 ▶); software used to prepare material for publication: *SHELXTL*.

## Supplementary Material

Crystal structure: contains datablocks global, I. DOI: 10.1107/S1600536810034604/wm2396sup1.cif

Structure factors: contains datablocks I. DOI: 10.1107/S1600536810034604/wm2396Isup2.hkl

Additional supplementary materials:  crystallographic information; 3D view; checkCIF report

## Figures and Tables

**Table 1:** Selected bond lengths (Å)

Cd1—N7	2.281 (2)
Cd1—N5	2.336 (2)
Cd1—N1	2.361 (2)
Cd1—N4	2.4004 (18)
Cd1—N2	2.406 (2)
Cd1—S2	2.6730 (8)

**Table 2:** Hydrogen-bond geometry (Å, °)

*D*—H⋯*A*	*D*—H	H⋯*A*	*D*⋯*A*	*D*—H⋯*A*
N3—H3⋯S1^i^	0.86	2.52	3.310 (2)	153
N6—H6⋯N8^ii^	0.86	2.14	2.958 (3)	159

Symmetry codes: (i) 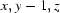; (ii) 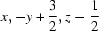.

**Fig. 1.**
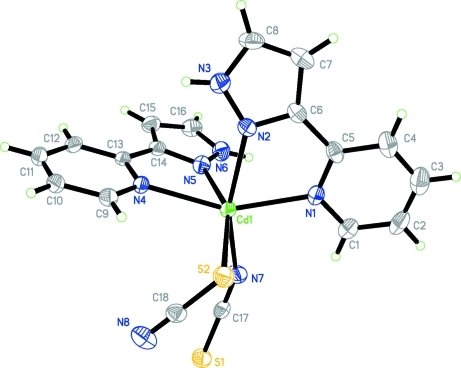
The molecular structure of compound (I) with atom labelling and displacement ellipsoids at the 30% probability level.

**Fig. 2.**
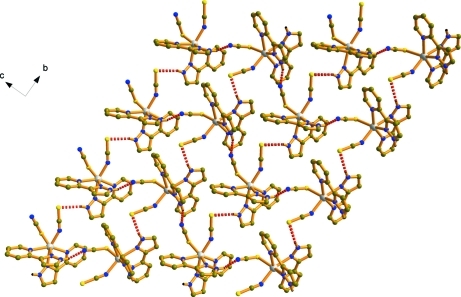
The sheet structure of compound (I), showing N—H···N and N—H···S hydrogen bongs as red dashed lines.

**Fig. 3.**
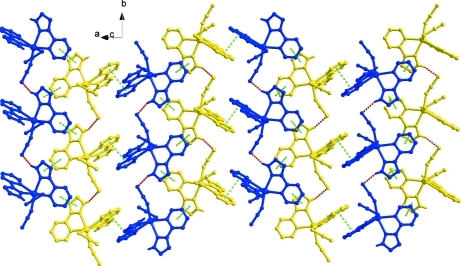
The three-dimensional packing of compound (I), showing π–π stacking as green dashed lines (red lines are hydrogen bonding interactions).

## supplementary crystallographic information

### Comment

Self-assembly processes directed by either hydrogen-bonding interactions or metal coordination have been extensively utilized in crystal engineering to construct supramolecular systems with novel structures and properties due to their inherent strength and reliability (Braga *et al.*, 2003; Chen & Liu, 2002; Zhang *et al.*, 2004). Proper selection of metal ions and ligands with suitable functionalized groups is the key issue in designing and self-assembling of molecules (Beatty, 2003). Very recently, we have initiated to utilize a multifunctional organic ligand, namely 3-(2-pyridyl)pyrazole (*L*), which acts as a simple bidentate chelate ligand, similar to 2,2'-bipyridine or 1,10-phenanthroline, to create a series of interesting metal-organic frameworks (Hu *et al.*, 2008). In the present paper, we report the crystal structure of the title compound (I), a new Cd^II^ complex based on the ligand *L* with additional SCN^-^ anions present.

In the molecular structure of the mononuclear complex (Fig. 1), the Cd^II^ atom is six-coordinated in a distorted octahedral geometry by five N atoms from one monodentate SCN^-^ anion and two bidentate chelating ligands *L*, and by one S atom from another SCN^-^ anion. The equatorial plane is defined by the SCN^-^ N atom, and three N atoms of the *L* ligands. The axial positions are occupied by one pyrazole N atom of a *L* ligand and the S atom the second SCN^-^ anion. The *L* ligand deviates slightly from planarity; the pyridyl and pyrazole rings make dihedral angles of 16.6 (2) and 3.3 (2)°, respectively. The *L* molecule adopts a bidentate chelate mode, in order to favor hydrogen bonding between the uncoordinated pyrazole N atoms and thiocyanate groups ligand. Each uncoordinated pyrazole N atom generates a hydrogen bond with two N and S atoms of the thiocyanate group. Furthermore, each complex is linked to four others, forming a (100) sheet, by N—H···N and N—H···S hydrogen bonding (Fig. 2). Face-to-face π-π stacking interactions between pyridyl-pyrazole and pyridyl-pyridyl rings link each sheet to two adjacent sheets, hence forming a three dimensional array (Fig. 3). The centroid-to-centroid distances between two neighboring almost parallel pyridyl-pyrazole rings are 3.805 (2) and 3.696 (2) Å, respectively.

### Experimental

Complex (I) was obtained by the reaction of Cd(NO_3_)_2_^.^4H_2_O, 3-(2-pyridyl)pyrazole (*L*) and NH_4_SCN in the molar ratio 1: 1: 1 in water (10 ml) under hydrothermal conditions at 393 K for three days. The autoclave was finally cooled down to room temperature at a rate of 5 Kh^-1^. The resulting solution was filtered and left to stand at room temperature. Colorless block-shaped crystals suitable for X-ray analysis were obtained in about 65% yield by slow evaporation of the solvent over a period of 1 week. Anal. calcd for C_18_H_14_CdN_8_S_2_: C,41.67; H,2.72, N, 21.59%; found: C, 41.63; H, 2.69; N, 21.54%.

### Refinement

Although all H atoms were visible in difference maps, they were finally placed in geometrically calculated positions, with C—H distances of 0.93Å and N—H distances of 0.86 Å, and included in the final refinement in the riding model approximation, with *U*_iso_(H) = 1.2*U*_eq_(C, N) for aromatic H atoms.

### Crystal data

**Table:**

[Cd(NCS)_2_(C_8_H_7_N_3_)_2_]	*F*(000) = 1032
*M**_r_* = 518.89	*D*_x_ = 1.687 Mg m^−^^3^
Monoclinic, *P*2_1_/*c*	Mo *K*α radiation, λ = 0.71073 Å
Hall symbol: -P 2ybc	Cell parameters from 4275 reflections
*a* = 14.4612 (19) Å	θ = 2.5–27.4°
*b* = 9.6043 (12) Å	µ = 1.30 mm^−^^1^
*c* = 14.9089 (19) Å	*T* = 296 K
β = 99.290 (2)°	Block, colourless
*V* = 2043.5 (5) Å^3^	0.32 × 0.26 × 0.22 mm
*Z* = 4	

### Data collection

**Table:**

Bruker APEXII CCD area-detector diffractometer	3602 independent reflections
Radiation source: fine-focus sealed tube	3119 reflections with *I* > 2σ(*I*)
graphite	*R*_int_ = 0.022
phi and ω scans	θ_max_ = 25.0°, θ_min_ = 2.5°
Absorption correction: multi-scan (*SADABS*; Bruker, 2003)	*h* = −17→9
*T*_min_ = 0.682, *T*_max_ = 0.764	*k* = −11→11
10166 measured reflections	*l* = −17→17

### Refinement

**Table:**

Refinement on *F*^2^	Primary atom site location: structure-invariant direct methods
Least-squares matrix: full	Secondary atom site location: difference Fourier map
*R*[*F*^2^ > 2σ(*F*^2^)] = 0.023	Hydrogen site location: inferred from neighbouring sites
*wR*(*F*^2^) = 0.053	H-atom parameters constrained
*S* = 1.05	*w* = 1/[σ^2^(*F*_o_^2^) + (0.0226*P*)^2^ + 0.5639*P*] where *P* = (*F*_o_^2^ + 2*F*_c_^2^)/3
3602 reflections	(Δ/σ)_max_ = 0.001
262 parameters	Δρ_max_ = 0.33 e Å^−^^3^
0 restraints	Δρ_min_ = −0.24 e Å^−^^3^

### Special details

**Table:**

Geometry. All e.s.d.'s (except the e.s.d. in the dihedral angle between two l.s. planes) are estimated using the full covariance matrix. The cell e.s.d.'s are taken into account individually in the estimation of e.s.d.'s in distances, angles and torsion angles; correlations between e.s.d.'s in cell parameters are only used when they are defined by crystal symmetry. An approximate (isotropic) treatment of cell e.s.d.'s is used for estimating e.s.d.'s involving l.s. planes.
Refinement. Refinement of *F*^2^ against ALL reflections. The weighted *R*-factor *wR* and goodness of fit *S* are based on *F*^2^, conventional *R*-factors *R* are based on *F*, with *F* set to zero for negative *F*^2^. The threshold expression of *F*^2^ > σ(*F*^2^) is used only for calculating *R*-factors(gt) *etc*. and is not relevant to the choice of reflections for refinement. *R*-factors based on *F*^2^ are statistically about twice as large as those based on *F*, and *R*- factors based on ALL data will be even larger.

### Fractional atomic coordinates and isotropic or equivalent isotropic displacement parameters (Å^2^)

**Table:**

	*x*	*y*	*z*	*U*_iso_*/*U*_eq_	
Cd1	0.229952 (12)	0.555357 (17)	0.337364 (11)	0.03567 (7)	
S1	0.32477 (5)	1.02032 (7)	0.45051 (5)	0.05433 (19)	
S2	0.12657 (5)	0.57892 (7)	0.46990 (5)	0.04796 (17)	
N1	0.10594 (14)	0.5820 (2)	0.21453 (13)	0.0402 (5)	
N2	0.18138 (14)	0.3329 (2)	0.27202 (13)	0.0413 (5)	
N3	0.21610 (16)	0.2024 (2)	0.27632 (15)	0.0510 (6)	
H3	0.2600	0.1737	0.3182	0.061*	
N4	0.34659 (14)	0.42207 (19)	0.43362 (13)	0.0359 (5)	
N5	0.36346 (15)	0.5293 (2)	0.26918 (14)	0.0419 (5)	
N6	0.38935 (16)	0.5695 (2)	0.19073 (14)	0.0481 (5)	
H6	0.3551	0.6189	0.1501	0.058*	
N7	0.25827 (17)	0.7890 (2)	0.34656 (15)	0.0552 (6)	
N8	0.26347 (18)	0.7180 (3)	0.59283 (16)	0.0653 (7)	
C1	0.06579 (19)	0.7044 (3)	0.19052 (18)	0.0494 (7)	
H1	0.0925	0.7850	0.2179	0.059*	
C2	−0.0137 (2)	0.7152 (3)	0.12667 (19)	0.0589 (8)	
H2	−0.0390	0.8020	0.1095	0.071*	
C3	−0.0551 (2)	0.5966 (4)	0.0887 (2)	0.0681 (9)	
H3A	−0.1102	0.6021	0.0470	0.082*	
C4	−0.0156 (2)	0.4704 (4)	0.11192 (18)	0.0590 (8)	
H4	−0.0435	0.3890	0.0868	0.071*	
C5	0.06664 (17)	0.4655 (3)	0.17354 (16)	0.0421 (6)	
C6	0.11593 (17)	0.3351 (3)	0.19786 (16)	0.0410 (6)	
C7	0.1093 (2)	0.2035 (3)	0.15527 (19)	0.0589 (8)	
H7	0.0693	0.1775	0.1027	0.071*	
C8	0.1745 (2)	0.1224 (3)	0.2080 (2)	0.0586 (8)	
H8	0.1874	0.0293	0.1981	0.070*	
C9	0.33719 (18)	0.3731 (2)	0.51578 (16)	0.0415 (6)	
H9	0.2809	0.3882	0.5368	0.050*	
C10	0.40707 (19)	0.3014 (3)	0.57064 (17)	0.0454 (6)	
H10	0.3983	0.2685	0.6273	0.054*	
C11	0.4903 (2)	0.2798 (3)	0.53915 (18)	0.0485 (7)	
H11	0.5389	0.2325	0.5749	0.058*	
C12	0.50132 (17)	0.3284 (2)	0.45472 (17)	0.0413 (6)	
H12	0.5573	0.3141	0.4329	0.050*	
C13	0.42789 (16)	0.3990 (2)	0.40266 (16)	0.0354 (5)	
C14	0.43478 (17)	0.4553 (2)	0.31247 (16)	0.0371 (6)	
C15	0.5074 (2)	0.4489 (3)	0.26009 (18)	0.0475 (6)	
H15	0.5648	0.4038	0.2746	0.057*	
C16	0.4754 (2)	0.5230 (3)	0.18351 (18)	0.0510 (7)	
H16	0.5072	0.5385	0.1350	0.061*	
C17	0.28474 (18)	0.8847 (3)	0.39008 (17)	0.0411 (6)	
C18	0.20761 (19)	0.6625 (3)	0.54154 (17)	0.0435 (6)	

### Atomic displacement parameters (Å^2^)

**Table:**

	*U*^11^	*U*^22^	*U*^33^	*U*^12^	*U*^13^	*U*^23^
Cd1	0.03330 (11)	0.03163 (10)	0.03974 (11)	0.00163 (8)	−0.00116 (7)	−0.00255 (8)
S1	0.0605 (5)	0.0379 (4)	0.0599 (4)	−0.0038 (3)	−0.0042 (4)	−0.0060 (3)
S2	0.0363 (4)	0.0557 (4)	0.0517 (4)	−0.0017 (3)	0.0065 (3)	−0.0062 (3)
N1	0.0355 (11)	0.0463 (13)	0.0389 (11)	0.0059 (10)	0.0061 (9)	0.0059 (9)
N2	0.0411 (12)	0.0385 (12)	0.0425 (12)	−0.0033 (10)	0.0012 (9)	−0.0049 (9)
N3	0.0541 (14)	0.0407 (13)	0.0558 (14)	0.0018 (11)	0.0014 (11)	0.0011 (11)
N4	0.0348 (11)	0.0334 (11)	0.0375 (11)	0.0022 (9)	−0.0002 (9)	0.0004 (8)
N5	0.0401 (12)	0.0405 (12)	0.0449 (12)	−0.0012 (10)	0.0063 (10)	0.0034 (9)
N6	0.0533 (14)	0.0481 (13)	0.0425 (12)	−0.0007 (11)	0.0067 (10)	0.0090 (10)
N7	0.0670 (16)	0.0360 (13)	0.0611 (15)	−0.0061 (12)	0.0062 (12)	−0.0068 (11)
N8	0.0662 (17)	0.0773 (18)	0.0530 (15)	−0.0144 (15)	0.0116 (13)	−0.0167 (13)
C1	0.0483 (16)	0.0484 (16)	0.0530 (16)	0.0079 (14)	0.0130 (13)	0.0077 (13)
C2	0.0558 (18)	0.070 (2)	0.0540 (17)	0.0284 (17)	0.0177 (14)	0.0211 (16)
C3	0.0472 (18)	0.105 (3)	0.0520 (18)	0.0130 (19)	0.0083 (14)	0.0102 (18)
C4	0.0439 (17)	0.086 (2)	0.0451 (16)	−0.0041 (16)	0.0013 (13)	0.0025 (15)
C5	0.0360 (14)	0.0607 (18)	0.0296 (12)	−0.0066 (13)	0.0052 (11)	0.0008 (12)
C6	0.0412 (15)	0.0465 (15)	0.0350 (13)	−0.0099 (12)	0.0050 (11)	−0.0022 (11)
C7	0.068 (2)	0.0604 (19)	0.0467 (16)	−0.0134 (17)	0.0038 (14)	−0.0142 (14)
C8	0.079 (2)	0.0358 (15)	0.0612 (18)	−0.0050 (16)	0.0124 (16)	−0.0120 (14)
C9	0.0458 (15)	0.0356 (13)	0.0418 (14)	−0.0009 (12)	0.0029 (11)	−0.0029 (11)
C10	0.0586 (18)	0.0360 (14)	0.0386 (14)	0.0024 (13)	−0.0011 (12)	0.0011 (11)
C11	0.0530 (17)	0.0361 (14)	0.0494 (16)	0.0057 (13)	−0.0131 (13)	−0.0012 (12)
C12	0.0359 (14)	0.0327 (13)	0.0529 (15)	0.0021 (11)	−0.0007 (11)	−0.0064 (11)
C13	0.0351 (13)	0.0251 (11)	0.0427 (13)	0.0004 (10)	−0.0037 (11)	−0.0081 (10)
C14	0.0370 (13)	0.0307 (13)	0.0423 (13)	−0.0029 (11)	0.0027 (11)	−0.0069 (11)
C15	0.0436 (15)	0.0455 (15)	0.0545 (16)	0.0038 (13)	0.0115 (13)	−0.0060 (13)
C16	0.0557 (18)	0.0499 (17)	0.0517 (17)	−0.0015 (14)	0.0213 (14)	−0.0015 (13)
C17	0.0398 (14)	0.0332 (13)	0.0495 (15)	0.0054 (12)	0.0052 (11)	0.0072 (12)
C18	0.0445 (15)	0.0479 (15)	0.0404 (14)	0.0025 (13)	0.0142 (12)	−0.0024 (12)

### Geometric parameters (Å, °)

**Table:**

Cd1—N7	2.281 (2)	C2—C3	1.367 (4)
Cd1—N5	2.336 (2)	C2—H2	0.9300
Cd1—N1	2.361 (2)	C3—C4	1.360 (4)
Cd1—N4	2.4004 (18)	C3—H3A	0.9300
Cd1—N2	2.406 (2)	C4—C5	1.381 (4)
Cd1—S2	2.6730 (8)	C4—H4	0.9300
S1—C17	1.636 (3)	C5—C6	1.458 (4)
S2—C18	1.660 (3)	C6—C7	1.410 (4)
N1—C1	1.335 (3)	C7—C8	1.368 (4)
N1—C5	1.355 (3)	C7—H7	0.9300
N2—C6	1.335 (3)	C8—H8	0.9300
N2—N3	1.348 (3)	C9—C10	1.378 (3)
N3—C8	1.339 (3)	C9—H9	0.9300
N3—H3	0.8600	C10—C11	1.376 (4)
N4—C9	1.339 (3)	C10—H10	0.9300
N4—C13	1.349 (3)	C11—C12	1.376 (4)
N5—C14	1.331 (3)	C11—H11	0.9300
N5—N6	1.341 (3)	C12—C13	1.387 (3)
N6—C16	1.342 (3)	C12—H12	0.9300
N6—H6	0.8600	C13—C14	1.468 (3)
N7—C17	1.154 (3)	C14—C15	1.408 (4)
N8—C18	1.150 (3)	C15—C16	1.362 (4)
C1—C2	1.372 (4)	C15—H15	0.9300
C1—H1	0.9300	C16—H16	0.9300
			
N7—Cd1—N5	88.76 (8)	C2—C3—H3A	120.0
N7—Cd1—N1	92.68 (8)	C3—C4—C5	118.7 (3)
N5—Cd1—N1	104.58 (7)	C3—C4—H4	120.6
N7—Cd1—N4	112.75 (7)	C5—C4—H4	120.6
N5—Cd1—N4	69.68 (7)	N1—C5—C4	121.7 (3)
N1—Cd1—N4	153.36 (7)	N1—C5—C6	116.4 (2)
N7—Cd1—N2	159.38 (7)	C4—C5—C6	121.9 (3)
N5—Cd1—N2	86.36 (7)	N2—C6—C7	110.3 (2)
N1—Cd1—N2	69.29 (7)	N2—C6—C5	118.2 (2)
N4—Cd1—N2	84.23 (6)	C7—C6—C5	131.5 (2)
N7—Cd1—S2	89.35 (6)	C8—C7—C6	105.1 (2)
N5—Cd1—S2	158.58 (5)	C8—C7—H7	127.4
N1—Cd1—S2	96.82 (5)	C6—C7—H7	127.4
N4—Cd1—S2	91.50 (5)	N3—C8—C7	107.1 (2)
N2—Cd1—S2	102.30 (5)	N3—C8—H8	126.5
C18—S2—Cd1	95.48 (9)	C7—C8—H8	126.5
C1—N1—C5	118.3 (2)	N4—C9—C10	123.1 (2)
C1—N1—Cd1	123.13 (17)	N4—C9—H9	118.5
C5—N1—Cd1	118.12 (15)	C10—C9—H9	118.5
C6—N2—N3	105.2 (2)	C11—C10—C9	118.1 (2)
C6—N2—Cd1	116.32 (16)	C11—C10—H10	121.0
N3—N2—Cd1	136.43 (16)	C9—C10—H10	121.0
C8—N3—N2	112.3 (2)	C12—C11—C10	119.9 (2)
C8—N3—H3	123.9	C12—C11—H11	120.1
N2—N3—H3	123.9	C10—C11—H11	120.1
C9—N4—C13	118.5 (2)	C11—C12—C13	119.1 (2)
C9—N4—Cd1	124.66 (16)	C11—C12—H12	120.5
C13—N4—Cd1	116.77 (15)	C13—C12—H12	120.5
C14—N5—N6	105.9 (2)	N4—C13—C12	121.3 (2)
C14—N5—Cd1	118.45 (16)	N4—C13—C14	116.4 (2)
N6—N5—Cd1	135.67 (16)	C12—C13—C14	122.3 (2)
N5—N6—C16	111.5 (2)	N5—C14—C15	110.1 (2)
N5—N6—H6	124.2	N5—C14—C13	118.7 (2)
C16—N6—H6	124.2	C15—C14—C13	131.2 (2)
C17—N7—Cd1	149.0 (2)	C16—C15—C14	105.0 (2)
N1—C1—C2	122.1 (3)	C16—C15—H15	127.5
N1—C1—H1	118.9	C14—C15—H15	127.5
C2—C1—H1	118.9	N6—C16—C15	107.5 (2)
C3—C2—C1	119.1 (3)	N6—C16—H16	126.2
C3—C2—H2	120.5	C15—C16—H16	126.2
C1—C2—H2	120.5	N7—C17—S1	178.5 (3)
C4—C3—C2	120.0 (3)	N8—C18—S2	178.3 (3)
C4—C3—H3A	120.0		
			
N7—Cd1—S2—C18	−51.87 (11)	N4—Cd1—N7—C17	−36.8 (5)
N5—Cd1—S2—C18	33.09 (17)	N2—Cd1—N7—C17	179.6 (3)
N1—Cd1—S2—C18	−144.49 (11)	S2—Cd1—N7—C17	54.5 (4)
N4—Cd1—S2—C18	60.87 (10)	C5—N1—C1—C2	0.5 (4)
N2—Cd1—S2—C18	145.29 (10)	Cd1—N1—C1—C2	−171.50 (19)
N7—Cd1—N1—C1	−14.5 (2)	N1—C1—C2—C3	2.4 (4)
N5—Cd1—N1—C1	−103.9 (2)	C1—C2—C3—C4	−2.4 (4)
N4—Cd1—N1—C1	−177.50 (17)	C2—C3—C4—C5	−0.4 (4)
N2—Cd1—N1—C1	175.8 (2)	C1—N1—C5—C4	−3.4 (4)
S2—Cd1—N1—C1	75.21 (19)	Cd1—N1—C5—C4	168.96 (19)
N7—Cd1—N1—C5	173.55 (18)	C1—N1—C5—C6	176.5 (2)
N5—Cd1—N1—C5	84.13 (18)	Cd1—N1—C5—C6	−11.2 (3)
N4—Cd1—N1—C5	10.5 (3)	C3—C4—C5—N1	3.4 (4)
N2—Cd1—N1—C5	3.83 (16)	C3—C4—C5—C6	−176.5 (3)
S2—Cd1—N1—C5	−96.78 (17)	N3—N2—C6—C7	0.3 (3)
N7—Cd1—N2—C6	−25.9 (3)	Cd1—N2—C6—C7	166.79 (17)
N5—Cd1—N2—C6	−102.57 (18)	N3—N2—C6—C5	−178.5 (2)
N1—Cd1—N2—C6	4.50 (16)	Cd1—N2—C6—C5	−12.0 (3)
N4—Cd1—N2—C6	−172.49 (18)	N1—C5—C6—N2	15.5 (3)
S2—Cd1—N2—C6	97.23 (17)	C4—C5—C6—N2	−164.6 (2)
N7—Cd1—N2—N3	135.1 (3)	N1—C5—C6—C7	−162.9 (3)
N5—Cd1—N2—N3	58.4 (2)	C4—C5—C6—C7	17.0 (4)
N1—Cd1—N2—N3	165.5 (2)	N2—C6—C7—C8	−0.1 (3)
N4—Cd1—N2—N3	−11.5 (2)	C5—C6—C7—C8	178.5 (3)
S2—Cd1—N2—N3	−101.8 (2)	N2—N3—C8—C7	0.4 (3)
C6—N2—N3—C8	−0.4 (3)	C6—C7—C8—N3	−0.2 (3)
Cd1—N2—N3—C8	−162.8 (2)	C13—N4—C9—C10	0.7 (3)
N7—Cd1—N4—C9	98.66 (19)	Cd1—N4—C9—C10	−177.48 (18)
N5—Cd1—N4—C9	178.3 (2)	N4—C9—C10—C11	0.2 (4)
N1—Cd1—N4—C9	−99.8 (2)	C9—C10—C11—C12	−0.6 (4)
N2—Cd1—N4—C9	−93.47 (18)	C10—C11—C12—C13	0.1 (4)
S2—Cd1—N4—C9	8.74 (18)	C9—N4—C13—C12	−1.1 (3)
N7—Cd1—N4—C13	−79.53 (17)	Cd1—N4—C13—C12	177.16 (16)
N5—Cd1—N4—C13	0.09 (15)	C9—N4—C13—C14	−179.73 (19)
N1—Cd1—N4—C13	82.1 (2)	Cd1—N4—C13—C14	−1.4 (2)
N2—Cd1—N4—C13	88.34 (16)	C11—C12—C13—N4	0.8 (3)
S2—Cd1—N4—C13	−169.45 (15)	C11—C12—C13—C14	179.3 (2)
N7—Cd1—N5—C14	116.27 (17)	N6—N5—C14—C15	0.1 (3)
N1—Cd1—N5—C14	−151.29 (16)	Cd1—N5—C14—C15	178.69 (15)
N4—Cd1—N5—C14	1.41 (16)	N6—N5—C14—C13	178.68 (19)
N2—Cd1—N5—C14	−83.78 (17)	Cd1—N5—C14—C13	−2.7 (3)
S2—Cd1—N5—C14	31.2 (3)	N4—C13—C14—N5	2.8 (3)
N7—Cd1—N5—N6	−65.6 (2)	C12—C13—C14—N5	−175.8 (2)
N1—Cd1—N5—N6	26.8 (2)	N4—C13—C14—C15	−179.0 (2)
N4—Cd1—N5—N6	179.5 (2)	C12—C13—C14—C15	2.4 (4)
N2—Cd1—N5—N6	94.3 (2)	N5—C14—C15—C16	0.0 (3)
S2—Cd1—N5—N6	−150.72 (17)	C13—C14—C15—C16	−178.4 (2)
C14—N5—N6—C16	−0.1 (3)	N5—N6—C16—C15	0.1 (3)
Cd1—N5—N6—C16	−178.39 (18)	C14—C15—C16—N6	−0.1 (3)
N5—Cd1—N7—C17	−104.2 (4)	Cd1—N7—C17—S1	107 (9)
N1—Cd1—N7—C17	151.3 (4)	Cd1—S2—C18—N8	−129 (9)

### Hydrogen-bond geometry (Å, °)

**Table:**

*D*—H···*A*	*D*—H	H···*A*	*D*···*A*	*D*—H···*A*
N3—H3···S1^i^	0.86	2.52	3.310 (2)	153
N6—H6···N8^ii^	0.86	2.14	2.958 (3)	159

Symmetry codes: (i) *x*, *y*−1, *z*; (ii) *x*, −*y*+3/2, *z*−1/2.
